# The cortico-rubral and cerebello-rubral pathways are topographically organized within the human red nucleus

**DOI:** 10.1038/s41598-019-48164-7

**Published:** 2019-08-20

**Authors:** Alberto Cacciola, Demetrio Milardi, Gianpaolo Antonio Basile, Salvatore Bertino, Alessandro Calamuneri, Gaetana Chillemi, Giuseppe Paladina, Federica Impellizzeri, Fabio Trimarchi, Giuseppe Anastasi, Alessia Bramanti, Giuseppina Rizzo

**Affiliations:** 1 Department of Biomedical, Dental Sciences and Morphological and Functional Images, University of Messina, Messina, Italy; 2 IRCCS Centro Neurolesi “Bonino Pulejo”, Messina, Italy

**Keywords:** Neural circuits, Neurological disorders

## Abstract

The Red Nucleus (RN) is a large nucleus located in the ventral midbrain: it is subdivided into a small caudal magnocellular part (mRN) and a large rostral parvocellular part (pRN). These distinct structural regions are part of functionally different networks and show distinctive connectivity features: the mRN is connected to the interposed nucleus, whilst the pRN is mainly connected to dentate nucleus, cortex and inferior olivary complex. Despite functional neuroimaging studies suggest RN involvement in complex motor and higher order functions, the pRN and mRN cannot be distinguished using conventional MRI. Herein, we employ high-quality structural and diffusion MRI data of 100 individuals from the Human Connectome Project repository and constrained spherical deconvolution tractography to perform connectivity-based segmentation of the human RN. In particular, we tracked connections of RN with the inferior olivary complex, the interposed nucleus, the dentate nucleus and the cerebral cortex. We found that the RN can be subdivided according to its connectivity into two clusters: a large ventrolateral one, mainly connected with the cerebral cortex and the inferior olivary complex, and a smaller dorsomedial one, mainly connected with the interposed nucleus. This structural topography strongly reflects the connectivity patterns of pRN and mRN respectively. Structural connectivity-based segmentation could represent a useful tool for the identification of distinct subregions of the human red nucleus on 3T MRI thus allowing a better evaluation of this subcortical structure in healthy and pathological conditions.

## Introduction

The red nucleus (RN) is a large neuronal structure located in the most rostral part of ventral midbrain. The name “red” is due to its high content of iron, which makes it clearly identifiable both in fresh sections and in T2-weighted MRI sequences. According to its cytoarchitecture, it can be divided in a rostral parvocellular part (pRN) and a caudal magnocellular part (mRN), both showing considerable variability in size and shape across different species^1^. From a phylogenetical perspective, the mRN is highly developed in quadrupedal mammals (i.e. rats or cats), it tends to become smaller in primates and to regress significantly in the human brain, where the pRN occupies most of the RN volume^2,3^. Along with such cytoarchitectonic differences, these two subregions significantly differ also in their connectivity patterns. Anatomical tract-tracing studies in primates demonstrated that the mRN receives its main afferences from the interposed nucleus^4,5^ and gives rise to the crossed rubrospinal tract, that reaches the spinal cord^6^. Conversely, the pRN receives afferents from the dentate nucleus^7,8^ and different regions of the cerebral cortex^3,9,10^ with the ipsilateral inferior olivary complex as main efference^11–13^. This different organization of connections likely reflects different functional roles. In quadrupedal animals, mRN exhibits a discharge pattern that is very similar to that of the pyramidal tract when avoiding obstacles during walking and is involved in intra and inter-limb coordination^14^. In monkeys, mRN activity, exerted through the stimulation of the rubrospinal tract, is involved in skilled movements such as grasping and reaching^15–17^. Finally, it has been postulated its potential in compensating lesions of the corticospinal tract^18,19^. By contrast, the pRN subserves different functional roles, the most of which largely remains still unknown. It has been suggested that it might participate in the regulation of the olivo-cerebellar system^20^, thus being involved in complex cognitive-motor functions such as motor learning, error encoding, timing and control of the ongoing movement^21^.

To date, just a few neuroimaging studies have been performed to investigate the human RN functional recruitment and connectivity. Results are very variable and suggest that the RN could be involved not only in motor control and execution but also in higher order sensory and cognitive functions, thus challenging the conceptions derived from animal literature^22^.

In a previous study, by using Constrained Spherical Deconvolution (CSD) signal modelling and probabilistic tractography, we described the  structural connectivity patterns of the RN with ipsilateral and contralateral dentate nucleus, the ipsilateral thalamus, cerebellar and cerebral cortex (superior frontal, precentral, postcentral and paracentral gyrus)^23^.

Since mRN and pRN are not distinguishable on conventional structural MRI scans, making inferences about which subregions of the RN were involved in the aforementioned studies is challenging.

Connectivity-based parcellation is a segmentation technique that uses connectivity data (e.g. derived from probabilistic tractography) to characterize the topographical organization of connections to given regions of interest^24^. The use of a “hypothesis-driven” approach applies prior anatomical knowledge about structural connections of a certain region of interest to subdivide it according to different connectivity targets^25^. Such approach can be easily applied to the study of the RN, since animal tract-tracing studies ascertain that the RN subregions can be distinguished according to their different connectivity patterns in (i) mRN, connected to the IN^5^ and (ii) pRN mainly connected with the dentate nucleus^7^, inferior olivary complex and cerebral cortex^3,26^.

The aim of the present study is to perform a structural connectivity-based parcellation of the RN in order to demonstrate that distinguishing between pRN and mRN in the human brain *in vivo* and non-invasively is feasible. RN connectivity patterns were reconstructed by means of CSD probabilistic tractography on high quality data from 100 healthy subjects of the Human Connectome Project repository^27^ and the RN was parcellated following a hypothesis-driven approach. We believe that unveiling the structural topographical organization of the RN may foster a better understanding of the functional anatomy of the human RN.

## Results

**Figure 1:**
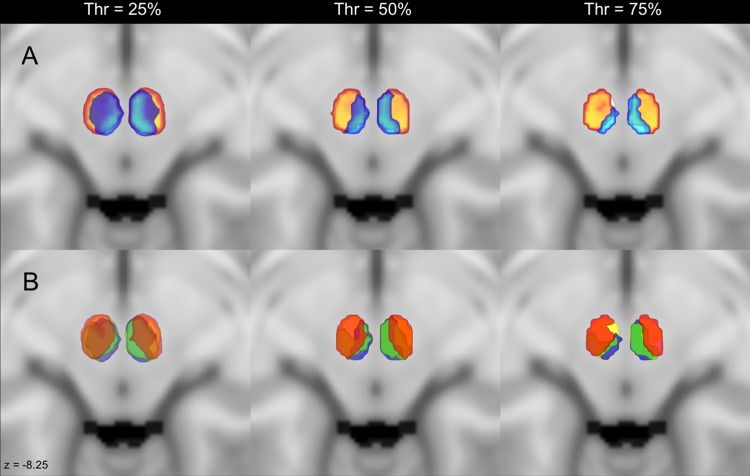
Comparison of maximum connectivity maps applying different thresholds. (**A**) Axial slices on the MNI152 template showing the topographical distribution of parvocellular (orange) and magnocellular (light blue) clusters when three different thresholds (25%, 50%, 75%) are applied. (**B**) Axial slices showing the topography of connectivity clusters of the cerebral cortex (red), inferior olivary complex (green), interposed nucleus (blue) and dentate nucleus (yellow) when the above-mentioned thresholds are applied. Note that the arrangements of the connectivity clusters are always maintained, even if higher thresholds lead to a cluster shrinkage.

**Figure 2:**
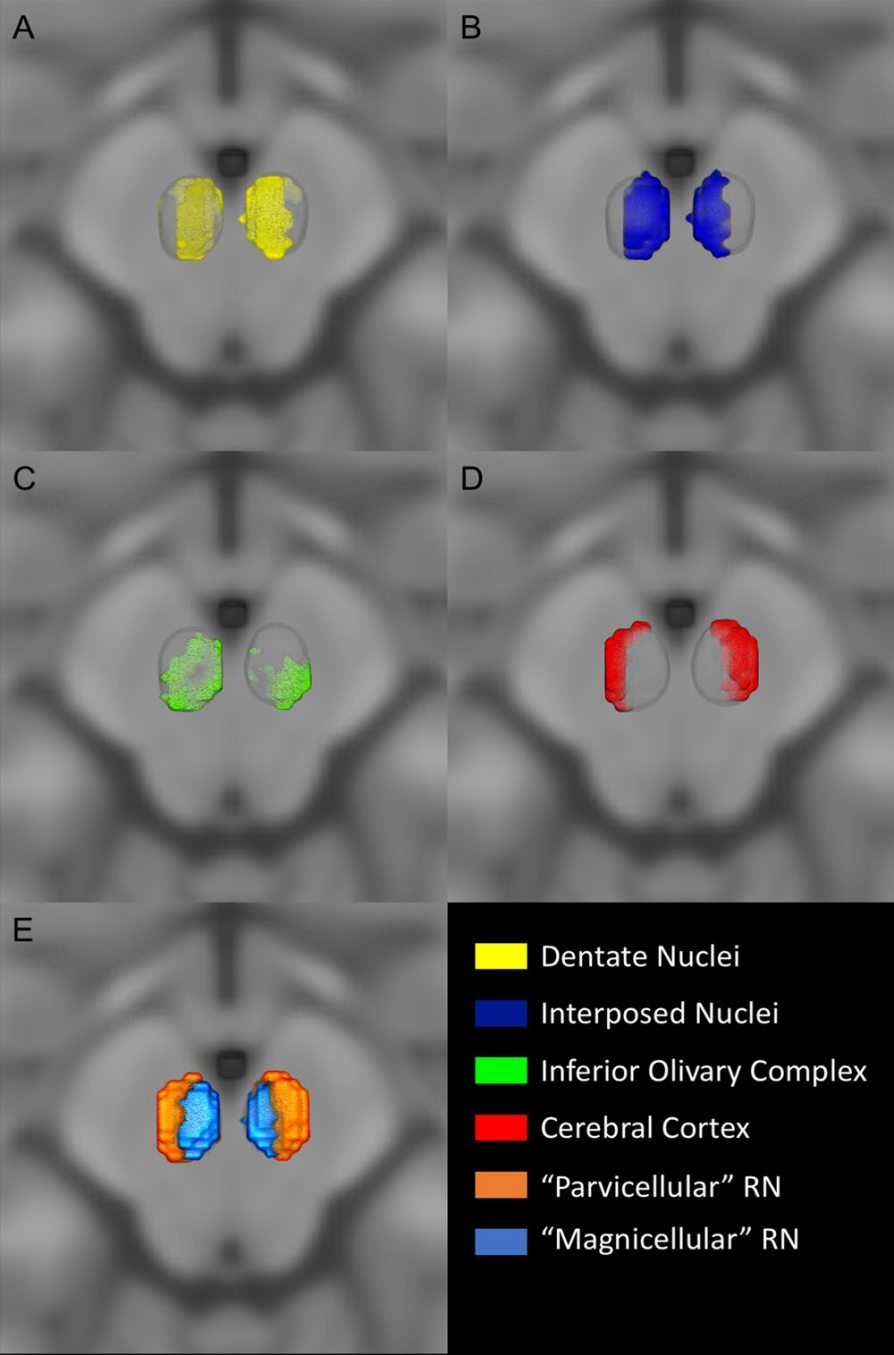
Connectivity clusters. Three-dimensional view of the identified connectivity clusters superimposed on the MNI152 space. MPM are thresholded at 75%. (**A**) Dentate nucleus (yellow) localized in the rostral and ventral portion of the red nucleus. (**B**) Interposed nucleus (green) clustered in the most caudal and medial part of the red nucleus. (**C**) Inferior olivary complex (green) occupied the most inferior region of the red nucleus. (**D**) Cerebral cortex (red) localized in the most lateral part of the red nucleus volume. (**E**) 3D visualization of parvocellular and magnocellular subregions on the MNI space, the parvocellular part is located in the ventrolateral aspect of the red nucleus while the magnocellular region is situated in its caudal and medial part.

**Figure 3:**
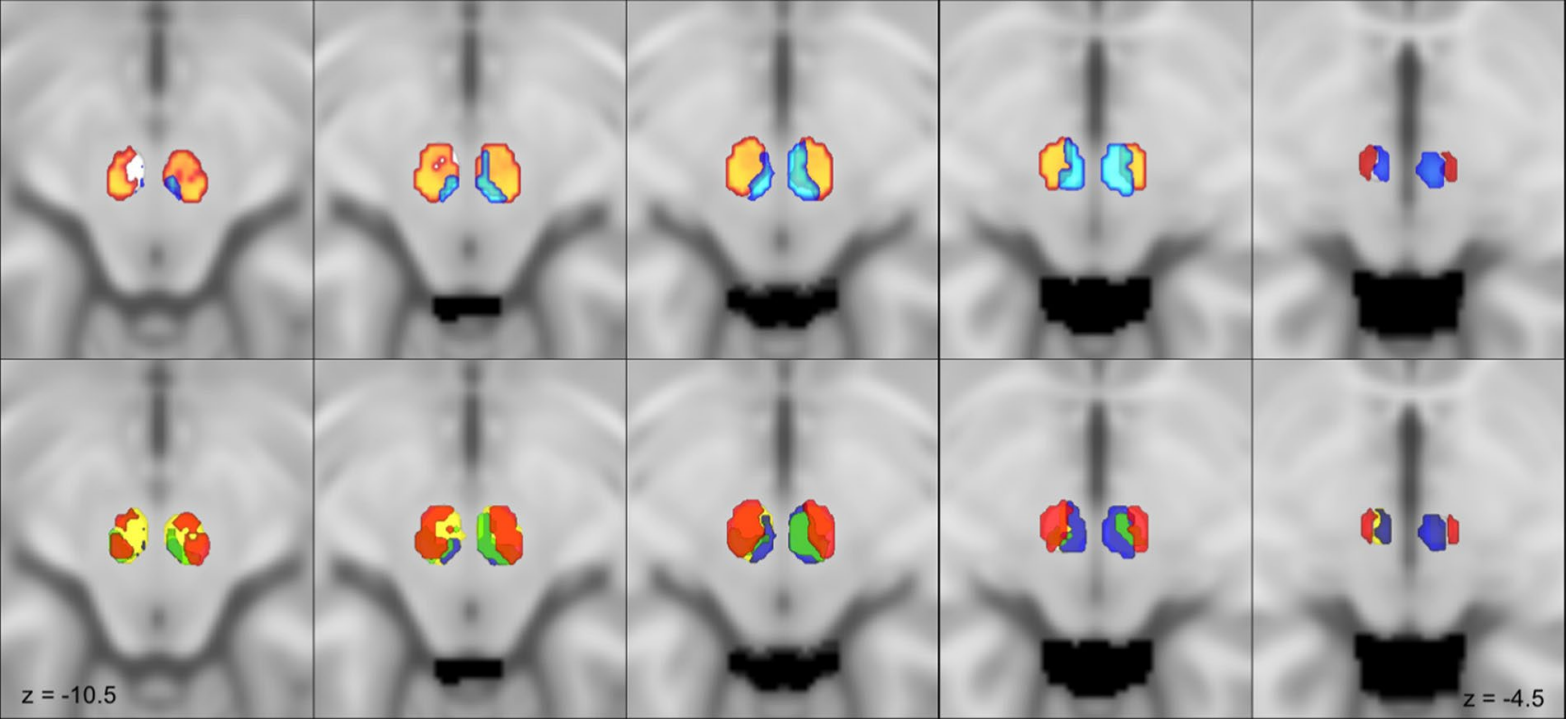
Red nucleus connectivity based parcellation. Multiple axial slices showing the identified connectivity clusters on the MNI152 template. MPM are thresholded at 75%. On the upper row the parvocellular (orange) and the magnocellular (light blue) clusters are shown; note that the magnocellular regions appears well delineated in the upper slices located dorsally while the parvocellular regions appears clearly from the second slides and quite disappears in the most cranial one. On the inferior row connectivity clusters for dentate nucleus (yellow), interposed nucleus (blue), inferior olivary complex (green) and cerebral cortex (red) are depicted. Transparency has been modulated to show the way different clusters overlap within red nucleus volume.

The arrangement of clusters, together with their respective Streamline Density Index (SDI) values (mean ± SD) are reported below and summarized in Table 1. Volumes and Centers of Gravity (CoG) are reported in Table 2. All clusters overlapped in more than 75% of subjects. Varying the threshold applied to each cluster’s Maximum Probability Map (MPM) did not modify the overall topographical arrangement, but just enlarged/reduced the coverage of each cluster (Fig. 1). For quantitative volume analysis and anatomical description, we considered then the 75%-thresholded MPM, which allows an optimal distinction of boundaries between clusters. The Lateralization Index for each pair of left and right clusters did not yield statistically significant results (p > 0.05). Figures 2 and 3 show all the target clusters respectively in 3D rendering and in multiple 2D axial sections.

**Table 1:** Volumes (mm^3^) and COG (x, y, z) of the connectivity clusters thresholded at 75% on the MNI152 space. Target regions are showed on the first column.

	LEFT		RIGHT	
	Volume (mm^3^)	COG (x, y, z)	Volume (mm^3^)	COG (x, y, z)
pRN	259	94.6, 106.24, 63.5	229	83.5, 106.6, 63.9
mRN	135	92.7, 105.8, 65	75	85.8, 105.7, 65.2
Dentate Nucleus	246	93.6, 106.2, 63.2	232	84.9, 106.4, 63.5
Cerebral Cortex	157	95.4, 106.4, 63.6	161	83.1, 106.9, 64.2
Interposed Nucleus	135	92.7, 105.8, 65	75	85.8, 105.7, 65.2
Inferior Olivary Complex	131	93.3, 105.3, 63.8	87	83.2, 104.7, 63.3

**Table 2:** Streamline density index (SDI) of the connectivity clusters thresholded at 75% expressed as mean (%) and related standard deviations (SD).

	LEFT		RIGHT	
	SDI (%)	SD	SDI (%)	SD
pRN	40	0.06	43.94	0.08
mRN	22.9	0.07	17.5	0.07
Dentate Nucleus	32.39	0.05	35.01	0.06
Cerebral Cortex	26.11	0.05	29.23	0.07
Interposed Nucleus	22.9	0.07	17.5	0.08
Inferior Olivary Complex	13.91	0.04	14.7	0.04

Target regions are showed on the first column.

### Target clusters

Each of the selected target clustered within the RN in a topographically organized manner.

Dentate nucleus (DN) clusters cover most of the whole RN volume (Fig. 2A), thus resulting in the highest SDI (Left = 32.39% ± 0.05; Right = 35.01% ± 0.06). DN cluster is mainly distributed in the rostral and ventral portions of RN (Fig. 3).

The interposed nucleus clustered mainly in the caudal and medial portions of the RN (Fig. 2B) (Left = 22.9% ± 0.07; Right = 17.5% ± 0.08). In axial sections, the interposed nucleus cluster is not visible in the most inferior sections; it appears ascending in an inferior-superior direction in the most caudal part and expands rostrally in the upper sections (Fig. 3).

The inferior olivary complex cluster is located in the most inferior portion of RN (Fig. 2C) (Left = 13.91% ± 0.04; Right = 14.07% ± 0.04), being well noticeable in the bottom sections while it gradually became less represented in the upper sections.

The cerebral cortex cluster extensively covers the RN volume and it occupies its lateral and anterior aspect (Fig. 2D) (Left = 26.11% ± 0.05; Right = 29.23% ± 0.07), in addition to be well appreciable and represented in almost every RN section (Fig. 3).

### Putative magnocellular and parvocellular clusters

Figure 2E represents the 3D rendering of the obtained putative magnocellular and parvocellular RN clusters. The parvocellular cluster is larger (Left = 40% ± 0.06; Right = 43.94% ± 0.08) and extends throughout the ventro-rostral region of the RN, while the magnocellular cluster shows lower SDI values (Left = 22.9% ± 0.07; Right = 17.5% ± 0.08) and it is located in the most caudal and dorsal aspect of RN. Ascending in infero-superior direction it occupies the most medial portion of RN and extends rostrally (Fig. 2E).

## Discussion

By using connectivity-based segmentation we demonstrated that the human RN is topographically organized according to its structural connectivity into two spatially distinguished clusters: a larger ventral one, mainly connected with the cerebral cortex and the inferior olivary complex, and a smaller dorsal one, mostly connected with the interposed nucleus. The dentate nucleus cluster did not show a clear topographic compartmentalization, covering mostly the entire volume of the RN. This result could be explained considering that the whole RN is traversed by the dento-thalamic decussating tract coming from the superior cerebellar peduncle and tractography is unable to clearly distinguish it from its dento-rubral collaterals^28^. Therefore, it is impossible to state whether the topography of the dentate cluster reflects the arrangement of direct dento-rubral connections rather than dento-thalamic fibers simply passing through RN. In addition, the rubrospinal connections were not taken into account in the present study, since it has been demonstrated that the decussating rubrospinal tract is very small and thin in the human brain^29^. This feature makes it difficult to be accurately reconstructed via probabilistic tractography^30^.

Taken together, the connectivity profiles reconstructed in the present study are in line with previous findings, emerged from tract tracing studies conducted in animals. Moreover, cortico-rubral and interpositus-rubral connections are topographically organized in the pRN and mRN respectively. Although the histological validation of this subdivision could be achieved only with post-mortem dissection and microscopy, our results could be interpreted at least as the possible evidence for the preservation of such a topographical segregation in the human RN.

The vast majority of the traditional literature assumes that only the pRN is structurally and functionally developed in humans, being the mRN only a vestigial, regressed and not clearly identifiable structure^31^. Contrasting this assumption, recent anatomical studies on human specimens described a small but well-delimitated magnocellular region within the RN, wrapped around the caudal pole of the pRN^3,32^. Our results are in line with these works showing that the connectivity patterns between the RN and the interposed nucleus clustered in the most caudal and dorsal portion of the RN, roughly corresponding to the mRN. Such cluster represents just a small portion (~20%) of the entire RN volume, the largest portion being mainly occupied by the dentate nucleus and cerebral cortex fibers, which should ideally represent the parvocellular cluster.

To the best of our knowledge, this work represents the first demonstration of the peculiar topographical arrangement of the RN in humans, by means of advanced tractography techniques. This could represent an important goal for further research, since animal studies have previously pointed out that such structural subdivisions could reflect very different functional roles. It is worthy to note that in humans most of the functions of RN are far from being fully understood.

The possible existence and functional activity of human mRN are supported by direct registration of neuronal activity of RN by means of microelectrodes in implanted patients. Rodriguez-Oroz and colleagues (2008) casually registered RN electrical activity in 2 Parkinsonian patients, due to the accidental misplacement of electrodes after deep brain stimulation of the adjacent subthalamic nucleus. The recorded RN firing pattern appears to be strongly related with both passive and active limb movements^33^; such a pattern is highly similar to that recorded in the monkey’s mRN^34^ and is then in strong contrast with the assumption of a complete functional regression of mRN, since, at least in monkeys, the pRN does not show an appreciable movement-related activity^5,35^.

Despite this, the effective functional meaning of this region is still unknown. Its main efference, the rubro-spinal tract, extends only to the first three cervical segments of the spinal cord in the adult human^29^. From an evolutionary point of view, regression of mRN in mammals is supposed to be related with the transition from quadrupedal to bipedal stance and with the subsequent loss of locomotor function in the upper limbs^1^. It has been then suggested that, paralleling phylogenesis, it may be involved in the acquisition of bipedal gait in the developing human brain^36^; and this would explain the observation of a well-developed mRN in human foetuses^37–40^.

On the other hand, the neuronal activity of mRN and rubrospinal neurons has been extensively characterized in primates. Indeed, it strongly relates with timing and magnitude of upper limb muscular activity^34,41–43^ and encodes both kinematic (velocity-related) and dynamic (force-related) parameters of upper limb movements^35,42,44^. More recent studies have suggested that, in monkeys, where upper limbs lose importance in locomotion and gain relevance for hand movements, grasping and manipulation, the mRN could be specialized in the execution of complex tasks requiring a hand pre-shape, such as reaching and grasping^17^. Although there is limited evidence supporting this view in humans, two different functional MRI (fMRI) studies aimed at investigating RN activity during active sensory discrimination, revealed RN activation during grasping, both when grasping was related to the sensory discrimination task and when it was not^45,46^.

Just a few neuroimaging studies directly focused on RN, and its functional role in the human brain remains broadly speculative. Task-related fMRI evidenced that RN was active during complex motor tasks such as grasping^45,46^, motor timing^47^, motor planning^48,49^ or movement initiation^50^. However, the RN also activates strongly during both passive tactile stimulation and active tactile discrimination^45^, as well as nociception, suggesting a possible role in somatic sensation^51,52^. Moreover, resting state functional connectivity pointed out that RN may be functionally connected not only with motor and premotor cortices, but also with higher-order cortices such as insula, prefrontal cortex, cingulate cortex, thus belonging to cognitive-affective functional networks (salience network, executive control network, default mode network)^22,53^. All the above-mentioned studies considered the RN as a whole, since distinguishing between mRN and pRN on conventional MRI scans is still a challenging issue. The present study proposes a structural connectivity-based segmentation approach as a useful tool to distinguish between putative mRN and pRN subregions by means of diffusion MRI and tractography in humans. Further investigations are then warranted to properly characterize the functional connectivity profile of RN subdivisions and their functional relevance in motor or cognitive tasks.

### Pathophysiological implications

Although their functional role remains still unclear, both subdivisions of the RN have been subject of interest by clinicians and researchers as they may play a role in the pathophysiology of different neurological disorders. The mRN and the rubrospinal tract are of notable interest in neurology since animal experiments have evidenced their role in compensating corticospinal tract in pyramidal lesions. Classical lesion experiments in monkeys showed that when the rubrospinal tract is selectively severed, the clinical outcome is a transient motor deficit, similar to a corticospinal tract lesion. Lesions in the rubrospinal system result in little or absent impairment when the corticospinal tract is intact, and, vice-versa, deficit from a corticospinal tract lesion are less severe and undergo an almost complete recover when the rubrospinal tract is intact. On the other hand, when both corticospinal and rubrospinal tracts are damaged, the deficit never recovers completely^54,55^. Corticospinal and rubrospinal neurons show significant similarities in electrophysiological properties, despite corticospinal output facilitates both proximal and distal upper limbs muscles^56^, whilst rubrospinal tract has stronger effects on distal muscles^16^. When the corticospinal tract is impaired, the rubrospinal tract undergoes plastic modifications and rearrangements in order to exert its facilitatory effect both on flexors and extensors^18^. In humans, there are sparse evidences for a similar compensation mechanism, mostly coming from diffusion imaging and tractography; however, recovery of pyramidal functions is slower and less complete than in monkeys. In chronic post-stroke patients, structural connectivity between primary and supplementary motor cortices and RN is significantly correlated with clinical measures of upper extremity functions^57^. Increased fractional anisotropy in RN, rubrospinal and corticorubral tracts was found to be positively correlated with the level of motor impairment in chronic post-stroke patients at different time intervals from lesion. Such changes in microstructural parameters have been interpreted as reflecting structural reorganization and brain plasticity^58–62^. These data suggest that the rubrospinal tract could have been underestimated, since it could have a great impact during neurorehabilitation. Our connectivity-based subdivision could help in understanding the functional role of RN subdivisions in stroke rehabilitation if combined with other techniques, such as fMRI.

Along with its implications in post-stroke recovery of pyramidal lesions, RN also seems to play an important role in the pathophysiology of tremors. A lesion in the dento-rubro-olivary loop (the so-called “Guilliain-Mollaret Triangle”) causes disinhibition in the inferior olivary firing pattern, resulting in hypertrophy of the inferior olive (hypertrophic olivary degeneration), that is clinically characterized by oculopalatal tremor^63^. Other clinical neurology findings are less significant, as lesions of the RN may potentially involve important adjacent structures such as the superior cerebellar peduncle, the subthalamic nucleus or the basal thalamus, thus leading to confounding clinico-pathological correlations^64^. The RN and its circuitry have been long-time considered to be involved in essential tremor (ET), a common neurological disease whose pathophysiological mechanisms are yet unknown. The classical paradigm of research, based on experimental models of harmaline-induced tremor in animals, suggests that tremors would originate from a dysregulation of the olivo-cerebellar firing^65–67^. Recent neuroimaging findings challenged this model and focused on neurodegenerative alterations of cerebello-thalamo-cortical systems^68–71^. Indeed, the RN is considered a key node in both the olivo-cerebellar and cerebello-thalamo-cortical systems and may be involved into ET pathogenesis. Supporting this view, Positron Emission Tomography (PET)^72,73^ and fMRI^74^ studies revealed abnormal RN activation in ET patients; in addition, alterations of diffusion parameters are also reported from diffusion tensor imaging studies^75,76^. The topographical organization revealed in the present study could be useful to shed new light on the pathophysiology of ET.

Moreover, recent findings suggest that the RN might be involved in Parkinson’s Disease, since several studies using advanced MRI techniques for iron detection and quantification reported progressive accumulation of iron in different brain nuclei including the RN itself. While only substantia nigra pars compacta is affected in the early stages of disease, substantia nigra pars reticulata, globus pallidus and RN are affected in advanced stages^77,78^. Iron content of RN, as measured by transverse relaxation rate (R2*), was correlated with development of levodopa-induced dyskinesia, thus reflecting a mechanism of cerebellar motor compensation after treatment with levodopa^79^. Another investigation correlated increased iron in the dentate nucleus and RN, measured with quantitative susceptibility mapping, with tremor symptoms in advanced PD patients^80^. Therefore, it would be interesting and challenging to investigate in PD patients the qualitative and quantitative changes of the RN topographical organization described in the present study.

All these evidences suggest the involvement of the RN in diseases of the motor system and emphasize the wide gap between basic and translational neuroscience. We hope our results could shed new light on RN functional anatomy in the human brain, moving towards a better understanding of its pathophysiological implications.

### Limitations

The main limitation of our study is related to intrinsic weaknesses of the technique employed. Along with its inability to detect signal directionality and presence of synapses^81,82^, and a recent paper underlined that tractographic reconstruction is prone to high rates of false positive tracts, when compared to ground truth tracts obtained from a simulated human brain dataset^83^. Despite its intrinsic limitations, tractography remains the only way to study structural connectivity in humans *in vivo*^84–97^. Moreover, it should be considered that the present study is based on a consolidated anatomical foundation and globally replicates what has already been found out in other species. Despite high quality diffusion MRI data and accurate pre-processing procedures have been employed, we cannot exclude that partial volume effects could introduce some degree of variability in our results. Considering that manual segmentation still represents the gold standard when it comes to ROI delineation, we believe that manual segmentation of RN on higher spatial resolution MRI acquisitions could better take into account the inter-individual variability of RN dimensions.

As many of the intrinsic limitations of the technique deal with streamline tracking and its sensitivity to local parameters, we believe that global tractography may provide a substantial improvement in further characterizing the structural topographical organization of the RN. Therefore, our next aim is to employ a global tractography approach to improve the accuracy, reducing sensitivity to local noise and modelling errors^98,99^. As regard the connectivity-based segmentation approach, we employed a hypothesis driven approach which derives each cluster from a predefined target. We are aware that this approach could potentially introduce a selection bias and our results would be more consistent if replicated by the application of a clustering algorithm, such as K-means, in a data-driven approach. However, we believe that a hypothesis-driven approach would fit better our purposes, since the connectivity-based subdivision into mRN and pRN is grounded on a-priori knowledge coming from a consistent background of animal research.

## Materials and Methods

### Subjects and data acquisition

High quality structural and diffusion MRI data of 100 healthy subjects (males = 46, females = 54 age range 22–36 years) from the Human Connectome Project (HCP) repository have been employed. Data were acquired by the Washington university, University of Minnesota and oxford university (WU-Minn) HCP Consortium^27^. All participants signed informed consent document at the day of scan as part of the Human Connectome Project, WU-Minn Consortium (Principal Investigators: David Van Essen and Kamil Ugurbil; 1U54MH091657) funded by the 16 NIH Institutes and Centers that support the NIH Blueprint for Neuroscience Research; and by the McDonnell Center for Systems Neuroscience at Washington University.” Subject recruitment procedures and informed consent forms, including consent to share de-identified data, were approved by the Washington University in St. Louis Institutional Review Board (IRB). All experimental procedures were performed under the guidelines of the HCP, which adhered to the relevant IRB processes related to that project; full details on the HCP have been published previously^100^.

The acquisitions were carried out using a Siemens 3T Skyra scanner previously modified with a Siemens SC72 gradient coil and stronger gradient power supply with maximum gradient amplitude (Gmax) of 100 mT/m (initially 70 mT/m and 84 mT/m in the pilot phase), thus allowing an improvement of diffusion imaging^101^. The structural scans included T1-weighted acquisitions with the following parameters: TE = 2.14 ms, TR = 2,400 ms, voxel size = 0.7 mm^101^. Diffusion-weighted images were acquired using a single-shot 2D spin-echo multiband Echo Planar Imaging (EPI) sequence and equally distributed over 3 shells (*b*-values of 1,000 s/mm^2^, 2,000 s/mm^2^, and 3,000 s/mm^2^), with isotropic spatial resolution of 1.25 mm^102^.

Data employed in this study were minimally pre-processed, thus normalization of b0 image intensity across runs and other corrections, such as those for EPI susceptibility, eddy-current-induced distortions, gradient-nonlinearities and subject motion were already carried out in the downloaded data^103^.

### MRI images post-processing

Both structural and diffusion images were post-processed in order to perform tractography. Briefly, structural images underwent brain extraction and cortical and subcortical segmentation^104–106^. The obtained masks were visually inspected and, if needed, modified by a trained neuroanatomist. A 5-tissue segmented image was then obtained and used to run multi-shell multi-tissue CSD (MSMT-CSD), an improvement of CSD signal modelling technique, in which fiber Orientation Distribution Function (fODF) is estimated directly from deconvolution of DW signal with a reference single fiber response function^107,108^. The MSMT-CSD modelling technique represents a variant designed to support multi-shell data and to overcome classical CSD limitations when it comes to estimate fODF in presence of tissue type heterogeneity^109^. Estimation of fODF and tractography were performed using the MrTrix software (www.mrtrix.org)^110^.

### Probabilistic tractography

A probabilistic whole-brain tractography of 5 million streamlines was run for each subject. Spherical harmonic degree was fixed equal to six to obtain robustness to noise. Tractography was performed with the following options: algorithm = *iFOD2*, step size = 0.2 mm, maximum angle = 10°, minimal fODF amplitude = 0.15.

### Region of interest (ROI) segmentation

Regions of interest (ROIs) for the red nucleus were obtained from the atlas provided by Keuken and Forstmann’s 7T atlas of the basal ganglia^111^. The same atlas was used to extract other midbrain regions (substantia nigra and periaqueductal gray) that could interfere with tractogram generation and selection leading to spurious results; such regions were thus used as regions of avoidance (ROAs). ROIs were warped from the MNI space to each subject’s native space using the following pipeline: first, an affine transformation was obtained by a linear registration algorithm (FLIRT); the affine transformation was used to obtain a nonlinear transformation (warp) from subject space to MNI space and vice-versa (inverse warp) by using FNIRT tool on FSL. The inverse warp was then applied to probabilistic ROIs in MNI space.

Deep cerebellar nuclei were segmented by using the SUIT Atlas, a probabilistic atlas of human cerebellum delineated in a dedicated Spatially Unbiased Infratentorial Template (SUIT); we opted for the use of a dedicated template, since it improves the alignment of infratentorial structures in respect to conventional MNI space^112–114^ The segmentation pipeline was run for each subject using the SUIT toolbox on SPM12^115^.

Cortical structures were extracted according to the Desikan-Killiany atlas featured in the Freesurfer software^116^.

**Figure 4:**
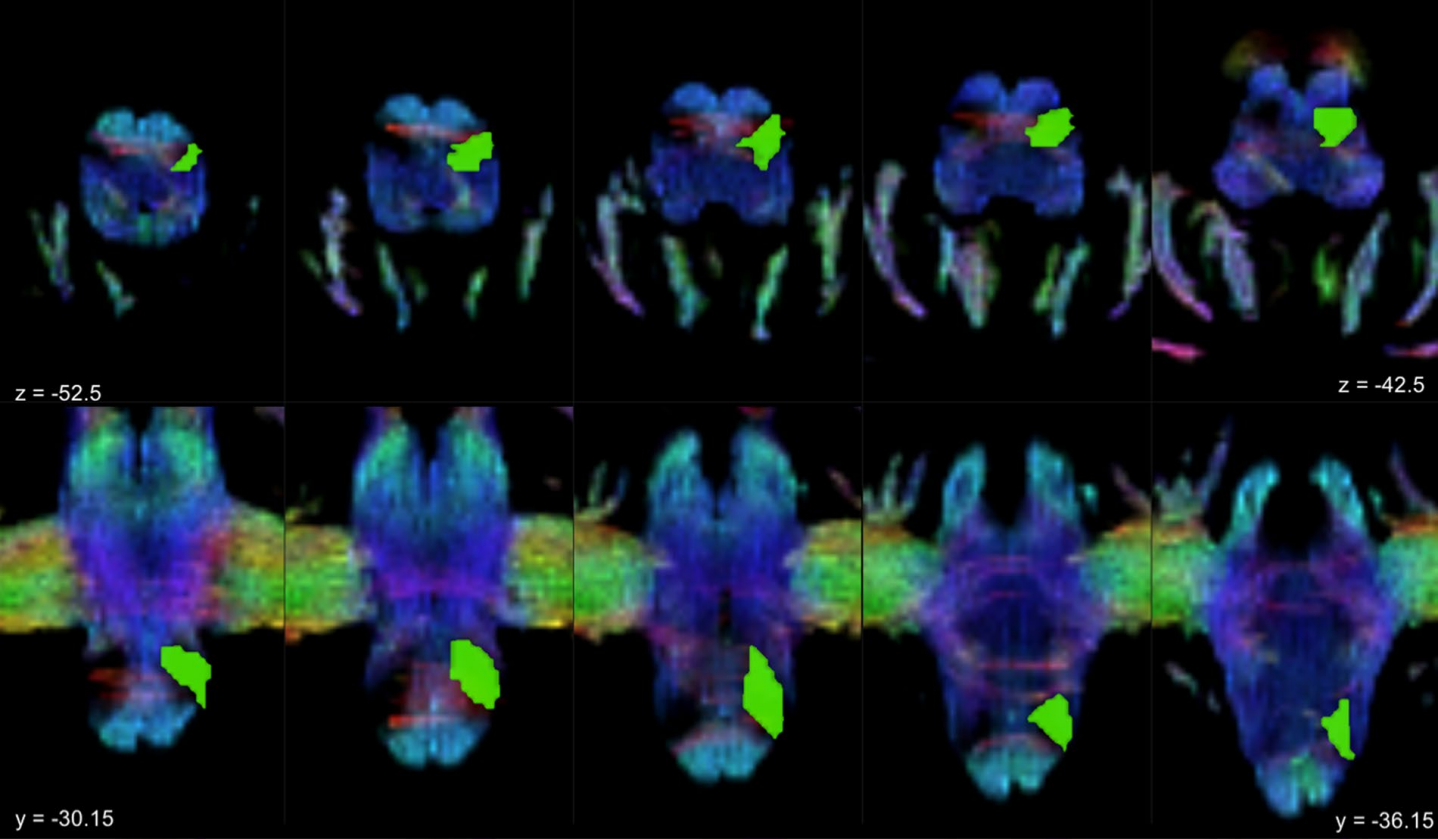
Inferior olivary complex manual segmentation. The figure shows a TDI (track density imaging) map of a sample subject. The superior row consists of axial slices where the inferior olivary complex ROI is superimposed on the left (green); a hypointense regions corresponding to the olivary complex can be observed on the right side just posterior to cortico-spinal tract. The same color-coding is maintained in the inferior row which consists of coronal slices showing the left ROI in green; notice that inferior olive is well contrasted from the surrounding white matter bundles.

As regard the inferior olivary complex, since, to the best of our knowledge, no brain atlases to segment this structure are available, we opted for a manual segmentation. On conventional MRI scans, boundaries of the inferior olivary complex are not clearly identifiable. Then, we adopted Track Density Imaging (TDI), an advanced structural imaging technique^117^ based on tractography that has been already used to successfully reconstruct major brainstem pathways^118^ and provides high anatomical accuracy for ROI placement^119^. From the 5-million-streamline tractogram a super-resolved map with 1 mm isotropic voxels was obtained. The intensity of each voxel was defined as the total number of streamlines passing within each super-resolution element^28,117,119^; maps were represented in directionally-encoded colours (DEC) to incorporate fiber directionality informations^118^. Inferior olivary complexes appeared then in the ventral medulla as an hypointense region postero-lateral to the hyperintense, blue color-coded (superior-inferior direction) pyramidal tracts. In each axial slice, pre-olivary and retro-olivary sulci marked their surface boundaries and olivary complexes appeared with a wedge shape with postero-medially directed vertices. For each subject, the ROIs of the inferior olivary complex were manually delineated bilaterally on axial sections by an experienced neuroanatomist, starting from the ponto-medullary junction and proceeding in a supero-inferior direction until the structures were not anymore identifiable (Fig. 4). A histological anatomic atlas^120^ was used as reference to individuate white matter tracts and structures. Boundaries were then checked and refined in coronal and sagittal sections.

### Connectivity-based parcellation

Connectivity-based parcellation was implemented using a hypothesis-driven approach at two different levels of analysis: at the first level, the RN was parcellated in clusters grouping voxels with highest connectivity with the four selected ROIs: dentate nucleus, interposed nucleus, inferior olivary complex and cerebral cortex. At the second level, clusters were grouped according to prior anatomical knowledge, attempting the identification of a putative magnocellular (mRN) and parvocellular (pRN) region basing on their different connectivity profiles. The dentate nucleus was excluded, due to the impossibility to distinguish between direct dentorubral fibers, that synapse in RN, and dentothalamic fibers that simply travel through the RN to reach thalamus. Hence, the putative magnocellular part was identified as the portion of RN with highest connectivity to the interposed nucleus, and the parvocellular one was identified as the portion of RN with highest connectivity to the cerebral cortex and inferior olivary complex.

Parcellation was implemented using the following pipeline:

1. Tracts between RN and the mentioned ROIs were extracted from the 5-million-streamlines tractogram obtained as above. The ROIs of contralateral RN, bilateral thalamus, periaqueductal gray and bilateral substantia nigra were used as exclusion masks to filter out spurious tracts. Since it is known that RN receives both ipsilateral and contralateral connections through the superior cerebellar peduncle, tracts between RN and both ipsilateral and contralateral deep cerebellar nuclei (dentate and interposed nuclei) were extracted.
2. Tracts were then converted into TDIs^117^, that were then mapped on the RN ROI to retrieve connectivity density-weighted clusters.
3. To reduce the potential effects of spurious tracking due to intrinsic tractography limitation, each cluster was then thresholded to 25% of its intensity.
4. Since we expected highest density values from highest connected clusters, each cluster was normalized by dividing its voxels’ intensity values by its mean intensity value, in order to obtain comparable connectivity density values.
5. A hard segmentation algorithm was used to compare between intensity values (find_the_biggest command in FSL). This algorithm retrieved as output, for each subject, a RN map in which each voxel is classified and attributed to the highest connected cluster (“winner takes all” method)^25^.
6. The obtained clusters were then mapped to the MNI152 standard space, binarized and summed up thus obtaining maximum probability maps (MPM) of average clusters between subjects.
7. Each MPM was finally thresholded to 25%, 50% and 75%.
8. For each of the obtained clusters, volumes and Center of Gravity (COG) in the MNI space were extracted from the 75%-thresholded MPM.

### Quantitative connectivity analysis

SDI was calculated as the percentage ratio between each cluster volume (in voxels) and the RN ROI volume^121^:

$$SDI=\frac{v}{{V}_{ROI}}\times 100$$

where *ν* is the volume (expressed in voxels) of each cluster and *V*_*ROI*_ is the volume (in voxels) of RN.

In addition, a lateralization index (LI)^122^ was calculated using the topographical maps volumes in subject space:

$$LI=\frac{Left-Right}{Left+Right}$$

Positive values of LI indicate left-lateralization (LI > 0.1), whereas negative values indicate right-lateralization (LI < 0.1). For each cluster, in order to assess statistically significant lateralization, permutation tests based on a t-statistic were performed using the connectivity profiles of each hemisphere gathered from each subject. 50,000 permutations were used to estimate the distribution of the null hypothesis, alpha level was set to 0.05, and the “t-max” method was adopted to correct the p-values of each variable for multiple comparisons^123^. LI analysis was performed by means of MATLAB software package (www.mathworks.com), release 2016.

## Data Availability

Data were provided by the Human Connectome Project (HCP); the Washington University, University of Minnesota, and Oxford University Consortium (Principal Investigators David Van Essen and Kamil Ugurbil; Grant No. 1U54MH091657) funded by 16 NIH institutes and centers that support the NIH Blueprint for Neuroscience Research; and the McDonnell Center for Systems Neuroscience at Washington University.
